# Supplementary material for: Posttranslational chemical installation of azoles into translated peptides
Source: Nat Commun. 2021 Jan 29;12:696. doi: 10.1038/s41467-021-20992-0 (PMC7846737; doi:10.1038/s41467-021-20992-0)
Supplement: Supplementary file 1 — Supplementary Information [file 41467_2021_20992_MOESM1_ESM.pdf]

Supplementary Information for:

Posttranslational chemical installation of azoles into translated peptides

Haruka Tsutsumi[1,†], Tomohiro Kuroda[1,†], Hiroyuki Kimura[1], Yuki Goto[1,*], and Hiroaki Suga[1,*]

Affiliation

[1]Department of Chemistry, Graduate School of Science, The University of Tokyo, Bunkyo, Tokyo 113-0033, Japan

[†] These authors contributed equally.

[*] Corresponding authors: Goto, Yuki (y-goto@chem.s.u-tokyo.ac.jp) and Suga, Hiroaki (hsuga@chem.s.u-tokyo.ac.jp)

Supplementary figures

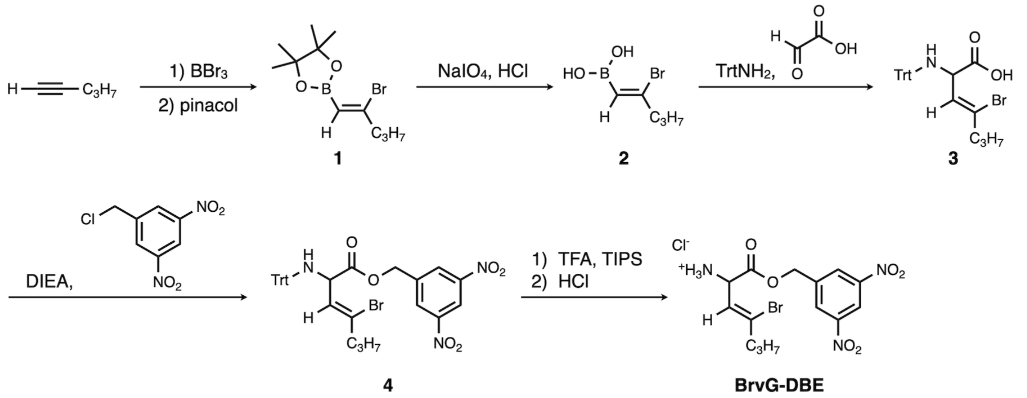

Supplementary Fig. 1. Synthetic scheme for **BrvG-DBE**.

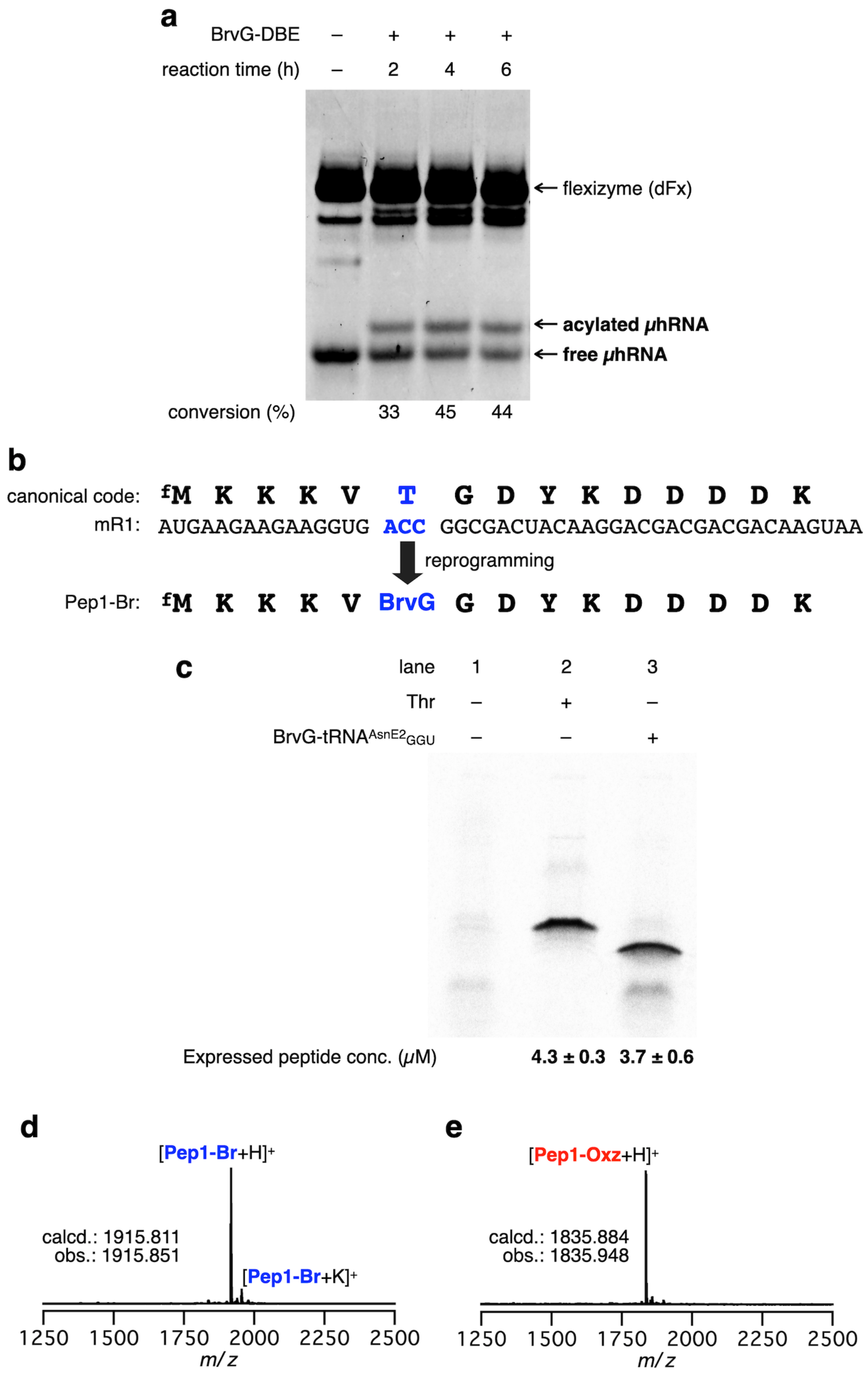

Supplementary Fig. 2. Ribosomal synthesis of peptides containing BrvG. (**a**) Aminoacylation of a tRNA analog with BrvG by flexizyme. A microhelix RNA (μhRNA) was incubated with BrvG-DBE in the presence of flexizyme for 2, 4, or 6 h, and analyzed by denaturing acid PAGE. The conversion yields of the aminoacylation were calculated

on the basis of the band intensities of the acylated μhRNA (I) and free μhRNA (II) as (I)/[(I) + (II)] × 100. The experiment was performed twice and a representative result was shown. The full range gel image is provided in **Supplementary Fig. 9a**. (**b**) Sequences of a model mRNA template (mR1) and its encoded peptide (Pep1-Br) in the reprogrammed FIT system. The ACC codon that canonically encodes Thr was reprogrammed with BrvG. (**c**) Tricine-SDS PAGE analysis of [^{14}C]-Asp-labelled translation products. Lane 1, background expression lacking Thr and BrvG-$tRNA^{AsnE2}_{GGU}$; lane 2, control expression by the canonical genetic code; lane 3, expression of Pep1-Br in the presence of BrvG- $tRNA^{AsnE2}_{GGU}$. The concentrations of the expressed peptides were determined, and the averaged values with standard deviations in triplicates were shown in the figure. A representative gel image in the triplicate experiments was shown. The full range gel image is provided in **Supplementary Fig. 9b**. (**d**) MALDI-TOF mass spectrum of Pep1-Br expressed in the reprogrammed FIT system. (**e**) MALDI-TOF mass spectrum of Pep1-Oxz generated by the posttranslational modification of BrvG by TBAF.

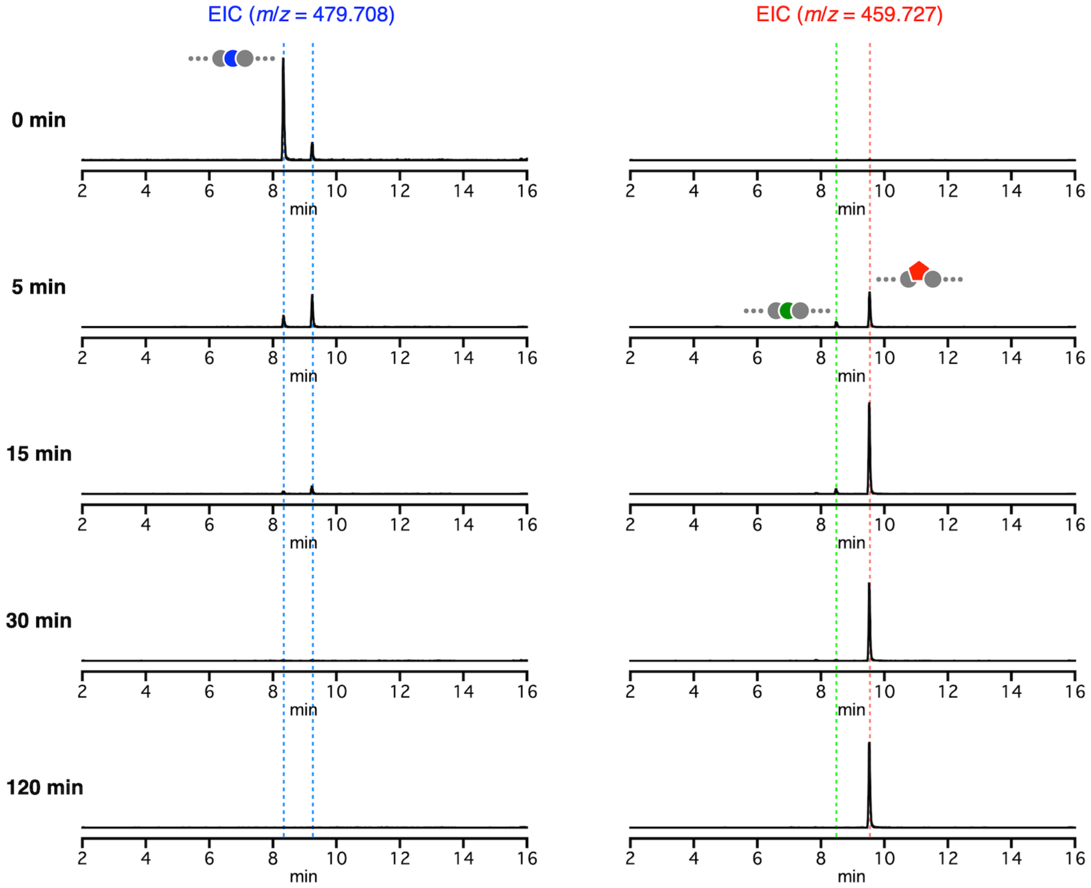

Supplementary Fig. 3. Additional EICs for the model chemical posttranslational modification of Pep1-Br at different time points. EICs of *m*/*z* values corresponding to the precursor (Pep1-Br/Pep1-Br’, 479.708) and the expected dehydrobrominated product (Pep1-AkyG/Pep1-Oxz, 459.727) are shown. The peaks corresponding to Pep1-Br/Pep1-Br’, Pep1-AkyG, and Pep1-Oxz are labelled with blue, green, and red dotted lines, respectively.

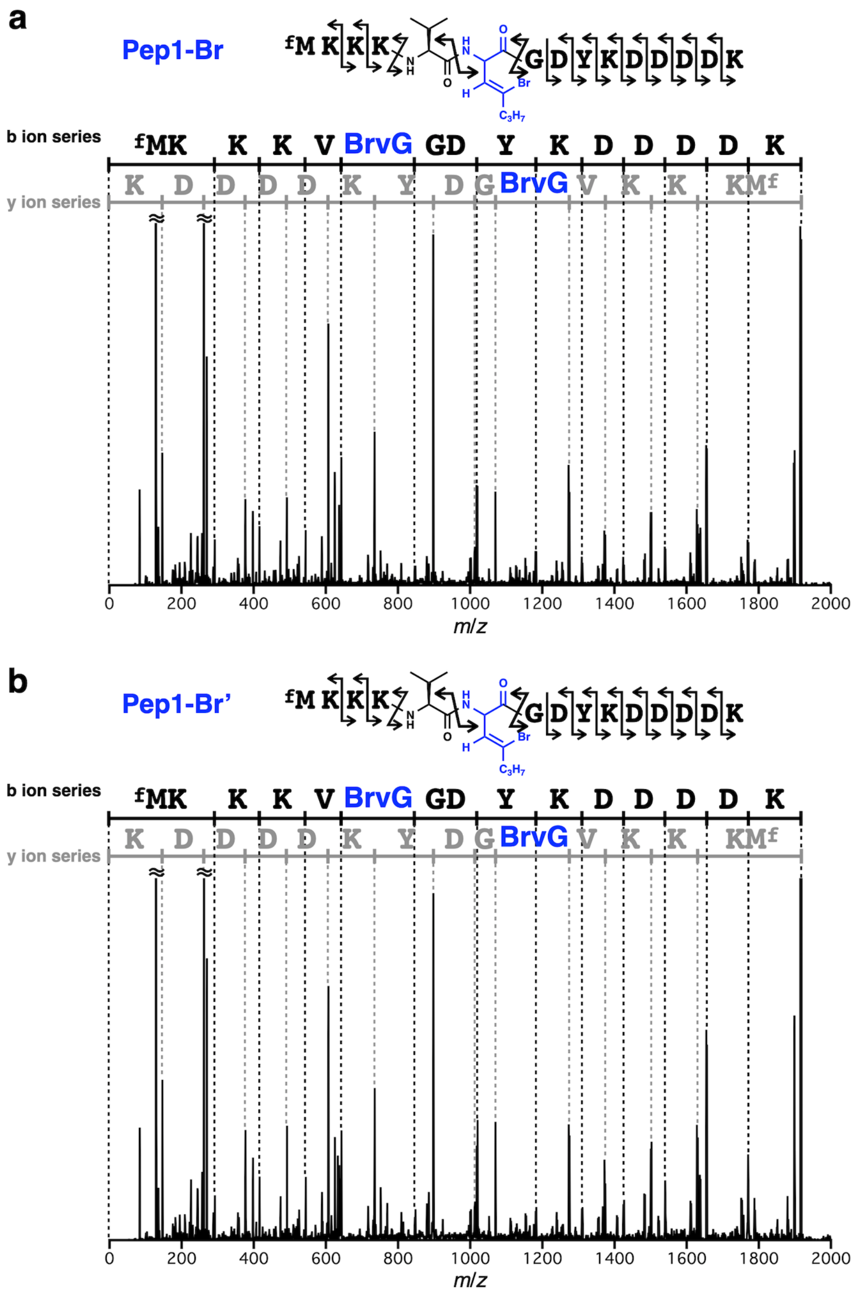

Supplementary Fig. 4. MS/MS spectra of (a) Pep1-Br and (b) Pep1-Br’. Observed b-ion series and y-ion series fragmentations are shown on the sequences in black and gray, respectively.

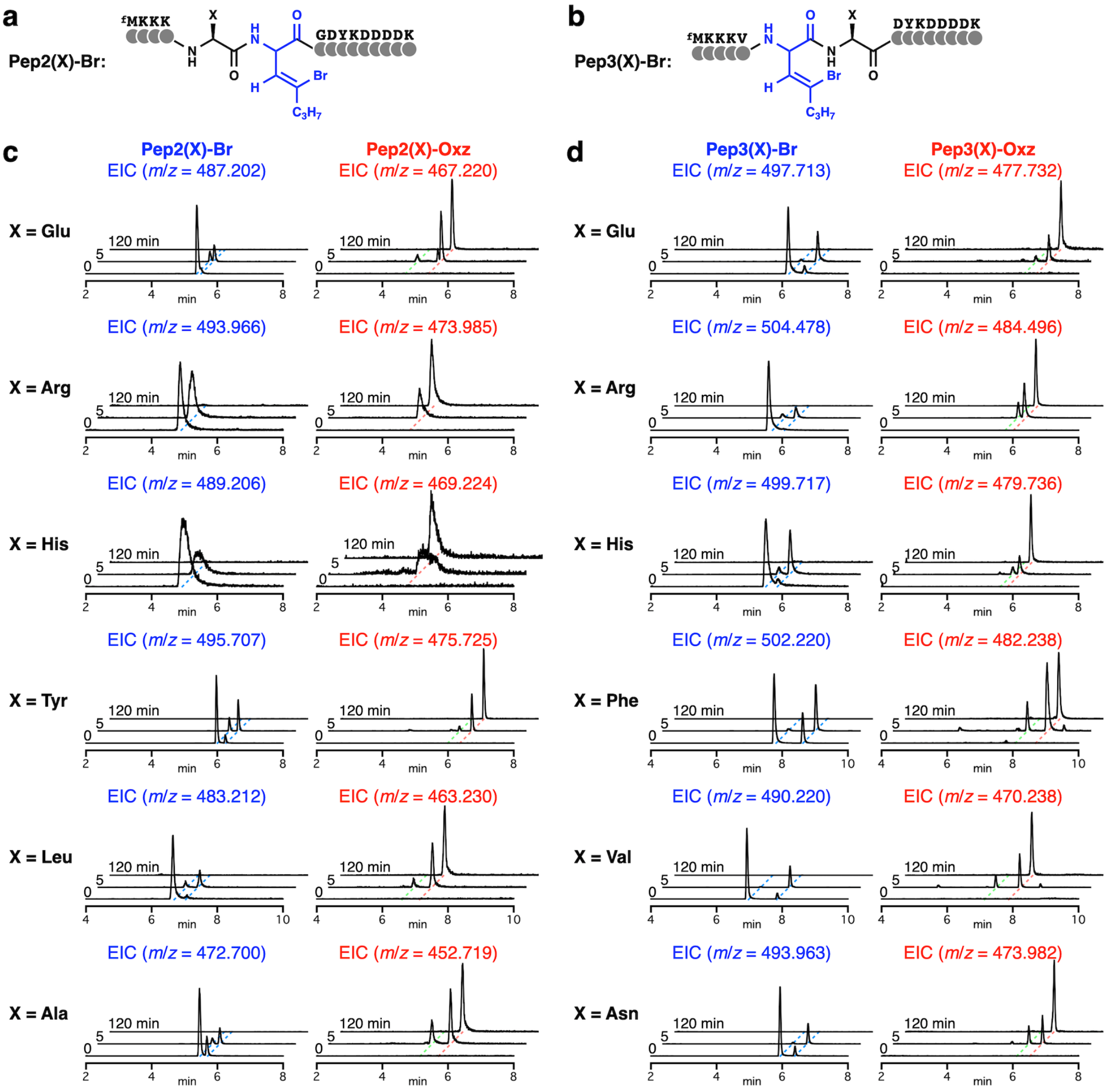

Supplementary Fig. 5. Chemical posttranslational modification of various BrvG-containing peptides. (**a**) Sequence of Pep2(X)-Br with different side chains at the upstream position of the BrvG. (**b**) Sequence of Pep3(X)-Br with different side chains at the downstream position of the BrvG. (**c**, **d**) Chemical posttranslational modification of various peptides with different side chains at the positions adjacent to the BrvG. EICs of *m*/*z* values corresponding to the precursor and the expected product are shown. In each panel, chromatograms of the samples after 0, 5, 120 min incubation are stacked. The peaks corresponding to peptides with BrvG, AkyG, and Oxz are labelled with blue, green, and red dotted lines, respectively.

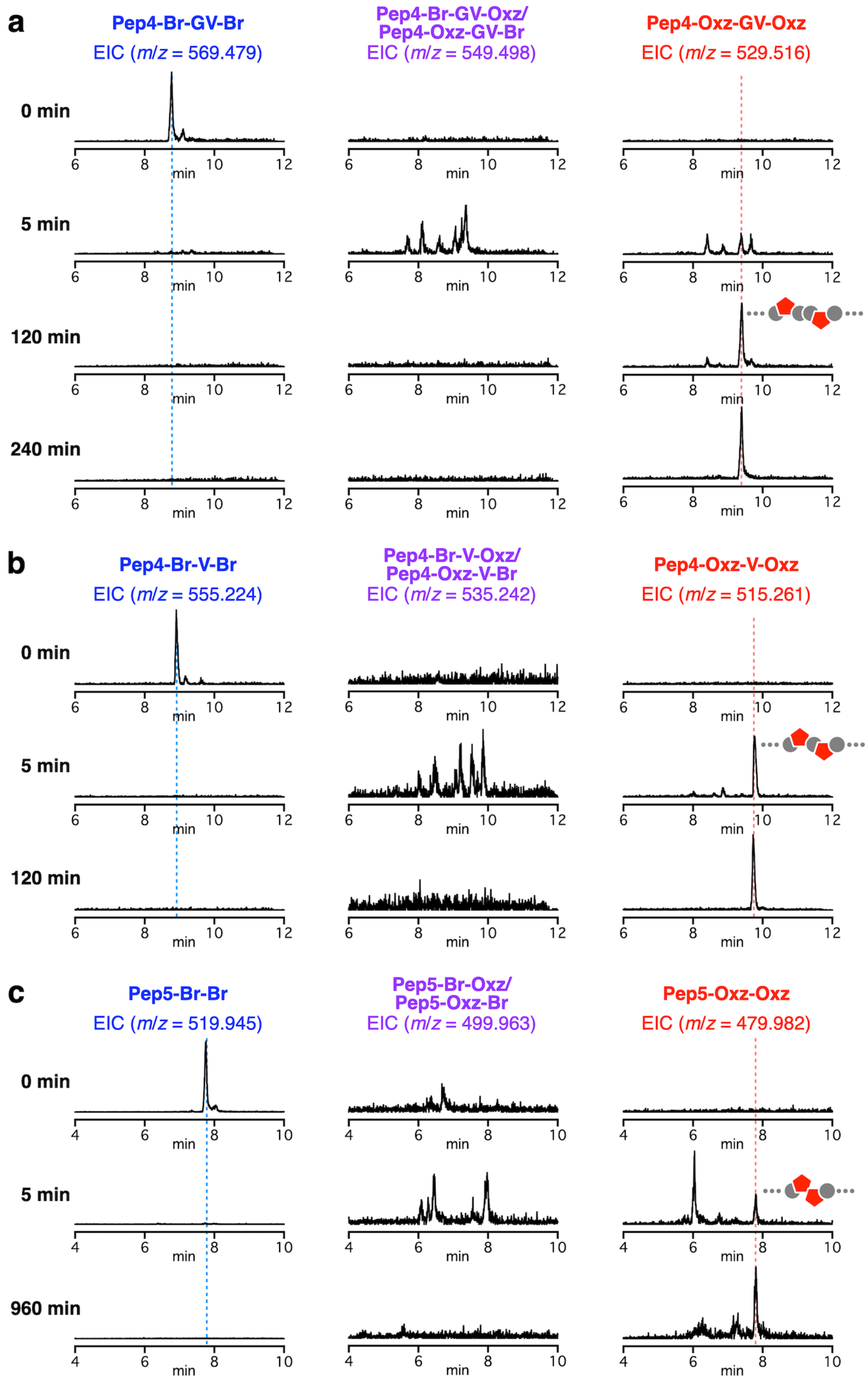

Supplementary Fig. 6. Chemical posttranslational modification of peptides containing two BrvG. (**a**–**c**) Chemical posttranslational modification of (**a**) Pep4-Br-GV-Br, (**b**) Pep4-Br-V-Br, and (**c**) Pep5-Br-Br. EICs of *m*/*z* values corresponding to the precursors bearing two BrvG, the intermediates bearing one BrvG and one oxazole, and the expected products bearing two oxazoles are shown.

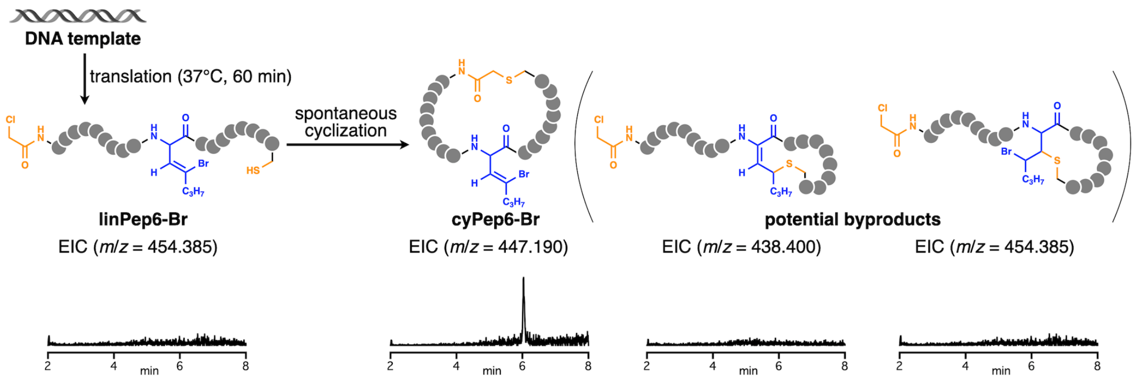

Supplementary Fig. 7. Ribosomal synthesis of the linear precursor (linPep6-Br) and subsequent spontaneous cyclization yielding the cyclic precursor (cyPep6-Br). EICs of *m*/*z* values corresponding to linPep6-Br, cyPep6-Br, and the expected potential byproducts of the spontaneous macrocyclization are shown. The EICs indicated that the expected cyclic precursor (cyPep6-Br) was selectively generated.

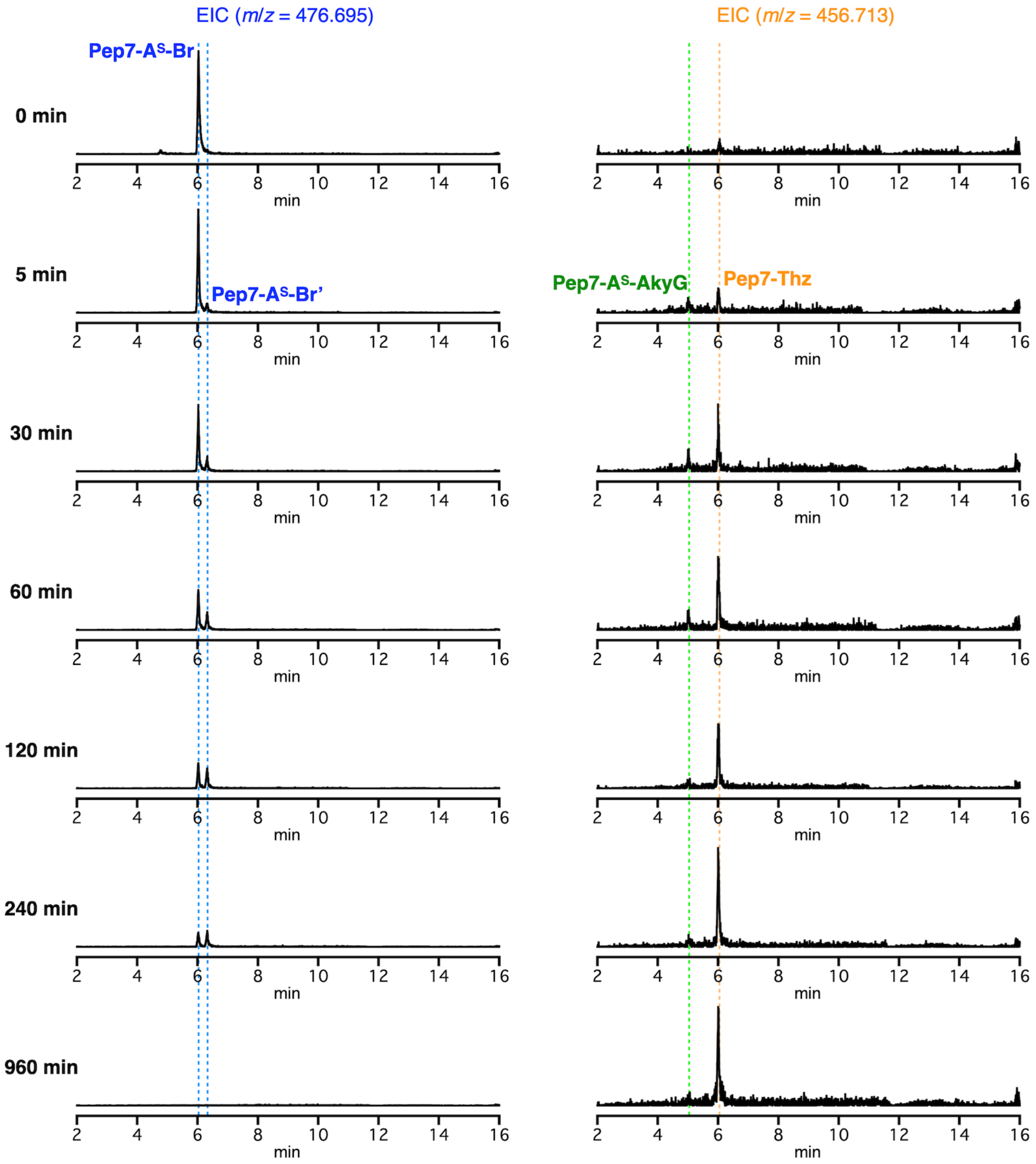

Supplementary Fig. 8. Additional EICs for the chemical posttranslational modification to yield the thiazole-containing peptide (Pep7-Thz). EICs of *m*/*z* values corresponding to the precursor Pep7-A^{S}-Br and the expected product Pep7-Thz are shown. The peaks corresponding to Pep7-A^{S}-Br/ Pep7-A^{S}-Br', Pep7-A^{S}- AkyG, and Pep7-Thz are labelled with blue, green, and orange dotted lines, respectively.

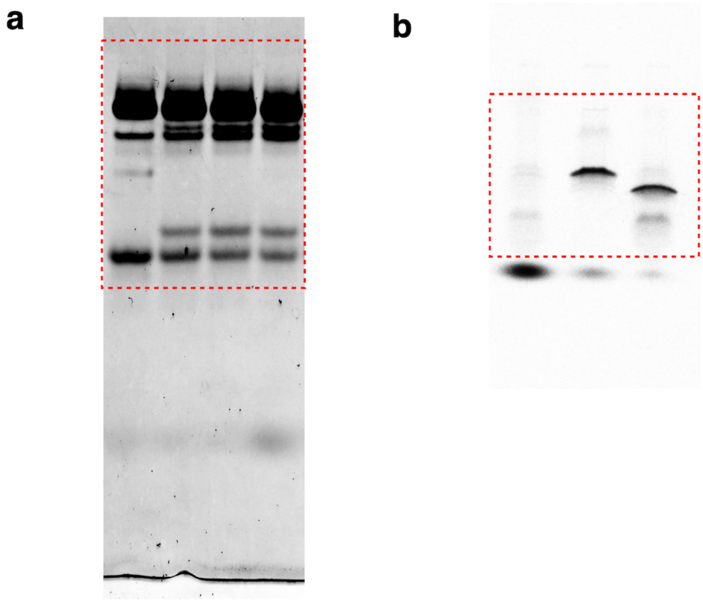

Supplementary Fig. 9. Full range gel images for the gel electrophoresis experiments performed in this study. (**a**) Denaturing acid PAGE of μhRNA. The range shown in **Supplementary Fig. 2a** is highlighted with red dotted rectangle. (**b**) Tricine-SDS PAGE of FIT-expressed Pep1-Br. The range shown in **Supplementary Fig. 2c** is highlighted with red dotted rectangle.

Supplementary methods

• Materials

All the chemical reagents were purchased from Cambridge Isotope Laboratories, Kanto Chemical, Nacalai Tesque, Sigma-Aldrich Japan, Tokyo Chemical Industry, Fujifilm Wako Pure Chemical Industries, or Watanabe Chemical Industries. All the chemical reagents were used without further purification. All the DNA oligomers were purchased from Eurofins Genomics.

• Chemical synthesis

(*Z*)-2-(2-bromopent-1-en-1-yl)-4,4,5,5-tetramethyl-1,3,2-dioxaborolane (1)[1]

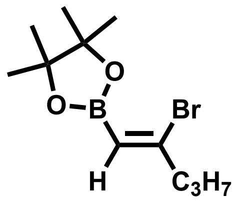

Under nitrogen atmosphere, a solution of boron tribromide (11 mL, 11 mmol, 1.0 M in DCM) was added to a solution of 1-pentyne (1.0 mL, 10.28 mmol) in DCM (5 mL) at -78°C. After stirring the mixture at -78°C for 2 h, pinacol (1.71 g, 14.5 mmol) was added to the solution. The reaction mixture was allowed to warm up to room temperature and stirred for 1 h. The mixture was poured into brine (50 mL) and extracted with DCM (30 mL×2). The combined organic layer was dried over Na_2SO_4, concentrated under reduced pressure, and purified by flash silica gel chromatography (EtOAc / hexane = 1/50) to yield **1** (929 mg, 33%) as an yellow oil.

^{1}H NMR (300 MHz, $CDCl_3$) δ 0.92 (3H, t, *J* = 7.5 Hz), 1.30 (12H, s), 1.59 (2H, sext, *J* = 7.4 Hz), 2.49 (2H, t, *J* = 7.4 Hz), 5.87 (1H, s). ^{13}C NMR (75 MHz, $CDCl_3$) δ 13.0, 21.3, 24.8, 47.3, 83.6, 144.9. HRMS-ESI (m/z) calcd for $C_{11}H_{20}BBrNaO_2^+$ ($[M+Na]^+$): 297.0632, found: 297.0627.

Synthesis of (*Z*)-(2-bromopent-1-en-1-yl)boronic acid (2)[2]

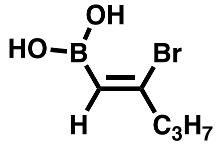

To a stirred solution of **1** (929 mg, 3.38 mmol) in THF (20 mL) was added H_2O (5 mL) and $NaIO_4$ (2.18 g, 10.1 mmol) at room temperature. After stirring the mixture for

30 min, HCl (0.23 mL, 0.23 mmol, 12 N) was added to the mixture and further stirred for 21 h. The mixture was diluted with water (30 mL) and the aqueous layer was extracted with EtOAc (60 mL×2). The combined organic layer was washed with water (30 mL×2) and brine (30 mL), dried over Na_2SO_4, and concentrated under reduced pressure to yield **2** (0.80 g, 77%) as an oil.

^{1}H NMR (300 MHz, $CDCl_3$) δ 0.93 (3H, t, *J* = 7.37 Hz), 1.57-1.69 (2H, m, *J* = 7.33 Hz), 2.54 (2H, dt, *J* = 0.78, 3.72 Hz), 5.17 (2H, br), 5.90 (1H, s). ^{13}C NMR (75 MHz, $CDCl_3$) δ 12.9, 21.2, 47.2, 146.2. HRMS-ESI (m/z) calcd for $C_5H_9BBrO_2^-$ ([M−H]$^-$): 190.9884, found: 190.9881

Synthesis of 3,5-dinitrobenzyl (*Z*)-4-bromo-2-(tritylamino)hept-3-enoate (4)[3]

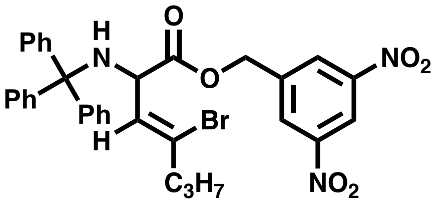

To a stirred solution of **2** (102 mg, 0.529 mmol) in PhMe (2.0 mL) was added glyoxylic acid monohydrate (49 mg, 0.530 mmol) and triphenylmethylamine (137 mg, 0.529 mmol) at room temperature. After 1 day, the mixture was filtered and the filterate was concentrated under reduced pressure. The obtained crude mixture was dissolved in 2.0 mL of $CHCl_3$ and 0.6 mL of the resulting solution was used for further reaction. After evaporating the crude material under reduced pressure, DMF (1.0 mL), 3,5-dinitrobenzyl chloride (100 mg, 0.477 mmol), and DIEA (81 µL, 0.477 mmol) were added and stirred at room temperature. After 25 h, the product was evaporated under reduced pressure and purified by silica gel chromatography (EtOAc / hexane = 1/8) to yield **4** (23 mg, 22%) as a solid.

^{1}H NMR (300 MHz, $CDCl_3$) δ 0.86-0.94 (3H, m), 1.56-1.66 (2H, m), 2.47 (2H, t, *J* = 7.07 Hz), 3.04 (1H d, *J* = 9.36 Hz), 4.40 (1H, t, *J* = 8.69 Hz), 4.57 (1H, d, *J* = 13.97 Hz), 4.95 (1H, d, *J* = 13.97 Hz), 5.73 (1H, d, *J* = 8.91 Hz), 7.17-7.29 (10H, m), 7.47-7.50 (6H, m), 8.34 (2H, d, *J* = 2.13 Hz), 8.95 (1H, t, *J* = 2.09 Hz). HRMS-ESI (m/z) calcd for $C_{33}H_{30}BrN_3NaO_6^+$ ([M+Na]$^+$): 666.1210, found: 666.1218.

Synthesis of 3,5-dinitrobenzyl (*Z*)-2-amino-4-bromohept-3-enoate (BrvG-DBE)

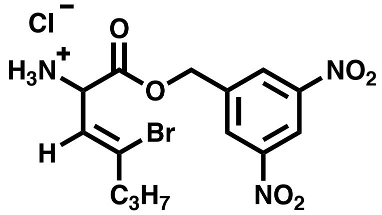

To a solution of **4** (15 mg, 0.023 mmol) in DCM (1.0 mL) was added 1.0 mL of TFA containing 10% TIPS at room temperature. After stirring the mixture for 20 min, it was diluted and coevaporated with Et_2O (10 mL×4). The product was dissolved in DCM (500 μL) and to this solution was added 0.4 M HCl in EtOAc (1.0 mL), which was immediately coevaporated (×3). The product was concentrated under reduced pressure and triturated from MeCN/EtOAc using Et_2O/hexane to yield **BrvG-DBE** (5.6 mg, 56%)

^{1}H NMR (300 MHz, CD_3CN) δ 0.86 (3H, t, J = 7.55 Hz), 1.58 (2H, sext, J = 7.1Hz), 2.54 (2H, t, J = 7.08 Hz), 5.06 (1H, d, J = 9.33 Hz), 5.51 (1H, d, J = 13.8 Hz), 5.58 (1H, d, J = 13.7 Hz), 6.16 (1H, d, J = 9.33 Hz), 8.62 (2H, d, J = 1.98 Hz), 8.86 (1H, t, J = 2.06 Hz). ^{13}C NMR (75 MHz, CD_3CN) δ 11.67, 20.59, 42.79, 54.28, 65.33, 118.16, 119.02, 127.75, 137.67, 139.19, 148.30, 166.43. HRMS-ESI (m/z) calcd for $C_{14}H_{17}BrN_3O_6^+$ ($[M-Cl]^+$): 402.0295, found: 402.0302.

• Preparation of DNA templates

DNA templates encoding each precursor peptide were prepared by PCR as previously described[4]. Primers used in the preparation of DNA templates and their sequences are listed in **Supplementary Tables 1** and **2**.

Supplementary Table 1. List of primer names used for the preparation of DNA templates coding the precursor peptide mutants.

DNA template name	Extension		PCR	
	forward primer	reverse primer	forward primer	reverse primer
mR1	T7eSD6M.F46	SD6MKKKV(ACC)Gflag.R48	T7ex5.F22	Flaguaa.R33
mR2(Glu)	T7eSD6M.F46	SD6MKKKE(ACC)Gflag.R48	T7ex5.F22	Flaguaa.R33
mR2(Arg)	T7eSD6M.F46	SD6MKKKR(ACC)Gflag.R48	T7ex5.F22	Flaguaa.R33
mR2(His)	T7eSD6M.F46	SD6MKKKH(ACC)Gflag.R48	T7ex5.F22	Flaguaa.R33
mR2(Tyr)	T7eSD6M.F46	SD6MKKKY(ACC)Gflag.R48	T7ex5.F22	Flaguaa.R33
mR2(Leu)	T7eSD6M.F46	SD6MKKKL(ACC)Gflag.R48	T7ex5.F22	Flaguaa.R33
mR2(Ala)	T7eSD6M.F46	SD6MKKKA(ACC)Gflag.R48	T7ex5.F22	Flaguaa.R33
mR3(Glu)	T7eSD6M.F46	SD6MKKKV(ACC)Eflag.R48	T7ex5.F22	Flaguaa.R33
mR3(Arg)	T7eSD6M.F46	SD6MKKKV(ACC)Rflag.R48	T7ex5.F22	Flaguaa.R33
mR3(His)	T7eSD6M.F46	SD6MKKKV(ACC)Hflag.R48	T7ex5.F22	Flaguaa.R33
mR3(Phe)	T7eSD6M.F46	SD6MKKKV(ACC)Fflag.R48	T7ex5.F22	Flaguaa.R33
mR3(Val)	T7eSD6M.F46	SD6MKKKV(ACC)Vflag.R48	T7ex5.F22	Flaguaa.R33
mR3(Asn)	T7eSD6M.F46	SD6MKKKV(ACC)Nflag.R48	T7ex5.F22	Flaguaa.R33
mR4(Gly-Val)	T7eSD6M.F46	SD6MKKKV(ACC)GV(ACC)Gflag.R57	T7ex5.F22	Flaguaa.R33
mR4(Val)	T7eSD6M.F46	SD6MKKKV(ACC)V(ACC)Gflag.R54	T7ex5.F22	Flaguaa.R33
mR5	T7eSD6M.F46	SD6MKKKG(ACCACC)Gflag.R51	T7ex5.F22	Flaguaa.R33
mR6	T7eSD6M.F46	mR6_a.R63	T7ex5.F22	mR6_b.R27
mR7	T7eSD6M.F46	SD6MKKKV(CACACC)Gflag.R51	T7ex5.F22	Flaguaa.R33

Supplementary Table 2. List of primer sequences used in this study.

primer name	sequence (5'- -3')
T7eSD6M.F46	TAATACGACTCACTATAGGGTTAACTTTAACAAGGAGAAAAACATG
SD6MKKKV(ACC)Gflag.R48	GTCGTCGTCCTTGTAGTCGCCGGTCACCTTCTTCTTCATGTTTTTCTC
T7ex5.F22	GGCGTAATACGACTCACTATAG
Flaguaa.R33	CGAAGCTTACTTGTCGTCGTCGTCCTTGTAGTC
SD6MKKKE(ACC)Gflag.R48	GTCGTCGTCCTTGTAGTCGCCGGTTTCCTTCTTCTTCATGTTTTTCTC
SD6MKKKR(ACC)Gflag.R48	GTCGTCGTCCTTGTAGTCGCCGGTACGCTTCTTCTTCATGTTTTTCTC
SD6MKKKH(ACC)Gflag.R48	GTCGTCGTCCTTGTAGTCGCCGGTATGCTTCTTCTTCATGTTTTTCTC
SD6MKKKY(ACC)Gflag.R48	GTCGTCGTCCTTGTAGTCGCCGGTGTACTTCTTCTTCATGTTTTTCTC
SD6MKKKL(ACC)Gflag.R48	GTCGTCGTCCTTGTAGTCGCCGGTCAGCTTCTTCTTCATGTTTTTCTC
SD6MKKKA(ACC)Gflag.R48	GTCGTCGTCCTTGTAGTCGCCGGTCGCCTTCTTCTTCATGTTTTTCTC
SD6MKKKV(ACC)Eflag.R48	GTCGTCGTCCTTGTAGTCCTCGGTCACCTTCTTCTTCATGTTTTTCTC
SD6MKKKV(ACC)Rflag.R48	GTCGTCGTCCTTGTAGTCTCTGGTCACCTTCTTCTTCATGTTTTTCTC
SD6MKKKV(ACC)Hflag.R48	GTCGTCGTCCTTGTAGTCGTGGGTCACCTTCTTCTTCATGTTTTTCTC
SD6MKKKV(ACC)Fflag.R48	GTCGTCGTCCTTGTAGTCGAAGGTCACCTTCTTCTTCATGTTTTTCTC
SD6MKKKV(ACC)Vflag.R48	GTCGTCGTCCTTGTAGTCCACGGTCACCTTCTTCTTCATGTTTTTCTC
SD6MKKKV(ACC)Nflag.R48	GTCGTCGTCCTTGTAGTCGTTGGTCACCTTCTTCTTCATGTTTTTCTC
SD6MKKKV(ACC)GV(ACC)Gflag.R57	GTCGTCGTCCTTGTAGTCGCCGGTCACGCCGGTCACCTTCTTCTTCATGTTTTTCTC
SD6MKKKV(ACC)V(ACC)Gflag.R54	GTCGTCGTCCTTGTAGTCGCCGGTCACGGTCACCTTCTTCTTCATGTTTTTCTC
SD6MKKKG(ACCACC)Gflag.R51	GTCGTCGTCCTTGTAGTCGCCGGTGGTGCCCTTCTTCTTCATGTTTTTCTC
mR6_a.R63	ACAGTTGTCGAACGGGTGGCCTCTGGTGTAGCCCGGTGCCTTTCTTGCGTGCATGTTTTTCTC
mR6_b.R27	CGAAGCTTAACAGTTGTCGAACGGGTG
SD6MKKKV(CACACC)Gflag.R51	GTCGTCGTCCTTGTAGTCGCCGGTGTGCACCTTCTTCTTCATGTTTTTCTC

• Preparation of flexizymes (dFx and eFx), $tRNA^{AsnE2}_{GGU}$, $tRNA^{fMet}_{CAU}$, and $tRNA^{GluE2}_{GUG}$

dFx, eFx, $tRNA^{AsnE2}_{GGU}$, $tRNA^{fMet}_{CAU}$, and $tRNA^{GluE2}_{GUG}$ were prepared by in vitro transcription with T7 RNA polymerase as previously described[4].

• Optimization of flexizyme-mediated aminoacylation conditions with BrvG-DBE

Reactions were carried out under the following conditions: 5 µL of 25 µM dFx, 25 µM µhRNA, and 5 mM **BrvG-DBE** in 0.1 M HEPES-K buffer pH 7.5, 600 mM $MgCl_2$ and 20% DMSO. After the reaction mixture was incubated on ice for 2–6 h, the reaction was stopped by the addition of 0.3 M sodium acetate (20 µL, pH 5.2) and 50 µL EtOH. The resulting mixture was centrifuged at 25°C, 15,300 ×g for 15 min and the supernatant was discarded. 25 µL of 70% EtOH that contained 0.1 M sodium acetate (pH 5.2) was added and centrifuged at 25°C, 15,300 ×g for 5 min. The supernatant was discarded and the pellet was air-dried for 5 min. The pellet was dissolved in 10 mM sodium acetate (1 µL, pH 5.2), and 15 µL of acid PAGE loading buffer (150 mM sodium acetate, 10 mM EDTA in 90% formamide) was added to this solution. 2 µL of the obtained mixture was applied to acid PAGE gel (20% acrylamide, 50 mM sodium acetate (pH 5.0), 6 M urea) and electrophoresis was carried out at 120 V for 2.5 h. The gel was stained with ethidium bromide, imaged by a Typhoon FLA 7000 (GE Healthcare) under control of FLA 7000 software v.1.2, and analyzed by ImageQuant TL v.8.1 (GE Healthcare).

• Preparation of aminoacylated tRNAs by flexizymes

BrvG, ^{ClAc}Y, and Ala^{S} were charged onto tRNA$^{AsnE2}_{GGU}$, tRNA$^{fMet}_{CAU}$, and tRNA$^{GluE2}_{GUG}$ by means of dFx, eFx, and dFx, respectively. Reactions were carried out under the following conditions: 5 µL of 25 µM dFx, 25 µM tRNA, and 5 mM acyl donor in 0.1 M HEPES-K buffer pH 7.5, 600 mM $MgCl_2$ and 20% DMSO. After the reaction mixture was incubated on ice for the optimized reaction time (4 h for BrvG, 2 h for ^{ClAc}Y, 0.5 h for Ala^{S}), the reaction was stopped by the addition of 0.3 M sodium acetate (20 µL, pH 5.2) and EtOH (50 µL). The resulting mixture was centrifuged at 25°C, 15,300 ×g for 15 min and the supernatant was discarded. The pellet was rinsed twice with 30 µL of 70% EtOH that contained 0.1 M sodium acetate (pH 5.2) and once with 70% EtOH. The resulting aminoacyl-tRNA was dissolved in 1 mM sodium acetate (0.5 µL, pH 5.2) just before adding to the translation mixture.

Supplementary Table 3. Summary of reaction conditions for in vitro translation carried out in this study.

peptide name	DNA template used	standard amino acids added (0.5 mM each)	precharged aminoacyl-tRNA added	reaction temperature	reaction time
Pep1-Br	mR1	D, G, K, M, V, Y	50 µM BrvG-tRNA$^{AsnE2}_{GGU}$	37°C	30 min
Pep2(Glu)-Br	mR2(Glu)	A, C, D, E, F, G, H, I, K, L, M, N, P, Q, R, V, W, Y	50 µM BrvG-tRNA$^{AsnE2}_{GGU}$	37°C	30 min
Pep2(Arg)-Br	mR2(Arg)	A, C, D, E, F, G, H, I, K, L, M, N, P, Q, R, V, W, Y	50 µM BrvG-tRNA$^{AsnE2}_{GGU}$	37°C	30 min
Pep2(His)-Br	mR2(His)	A, C, D, E, F, G, H, I, K, L, M, N, P, Q, R, V, W, Y	50 µM BrvG-tRNA$^{AsnE2}_{GGU}$	37°C	30 min
Pep2(Tyr)-Br	mR2(Tyr)	A, C, D, E, F, G, H, I, K, L, M, N, P, Q, R, V, W, Y	50 µM BrvG-tRNA$^{AsnE2}_{GGU}$	37°C	30 min
Pep2(Leu)-Br	mR2(Leu)	A, C, D, E, F, G, H, I, K, L, M, N, P, Q, R, V, W, Y	50 µM BrvG-tRNA$^{AsnE2}_{GGU}$	37°C	30 min
Pep2(Ala)-Br	mR2(Ala)	A, C, D, E, F, G, H, I, K, L, M, N, P, Q, R, V, W, Y	50 µM BrvG-tRNA$^{AsnE2}_{GGU}$	37°C	30 min
Pep3(Glu)-Br	mR3(Glu)	A, C, D, E, F, G, H, I, K, L, M, N, P, Q, R, V, W, Y	50 µM BrvG-tRNA$^{AsnE2}_{GGU}$	37°C	30 min
Pep3(Arg)-Br	mR3(Arg)	A, C, D, E, F, G, H, I, K, L, M, N, P, Q, R, V, W, Y	50 µM BrvG-tRNA$^{AsnE2}_{GGU}$	37°C	30 min
Pep3(His)-Br	mR3(His)	A, C, D, E, F, G, H, I, K, L, M, N, P, Q, R, V, W, Y	50 µM BrvG-tRNA$^{AsnE2}_{GGU}$	37°C	30 min
Pep3(Phe)-Br	mR3(Phe)	A, C, D, E, F, G, H, I, K, L, M, N, P, Q, R, V, W, Y	50 µM BrvG-tRNA$^{AsnE2}_{GGU}$	37°C	30 min
Pep3(Val)-Br	mR3(Val)	A, C, D, E, F, G, H, I, K, L, M, N, P, Q, R, V, W, Y	50 µM BrvG-tRNA$^{AsnE2}_{GGU}$	37°C	30 min
Pep3(Asn)-Br	mR3(Asn)	A, C, D, E, F, G, H, I, K, L, M, N, P, Q, R, V, W, Y	50 µM BrvG-tRNA$^{AsnE2}_{GGU}$	37°C	30 min
Pep4-Br-GV-Br	mR4(Gly-Val)	D, G, K, M, V, Y	100 µM BrvG-tRNA$^{AsnE2}_{GGU}$	37°C	30 min
Pep4-Br-V-Br	mR4(Val)	D, G, K, M, V, Y	100 µM BrvG-tRNA$^{AsnE2}_{GGU}$	37°C	30 min
Pep5-Br-Br	mR5	A, C, D, E, F, G, H, I, K, L, M, N, P, Q, R, V, W, Y	100 µM BrvG-tRNA$^{AsnE2}_{GGU}$	37°C	30 min
linPep6-Br	mR6	A, C, D, E, F, G, H, I, K, L, N, P, Q, R, V, W, Y	50 µM BrvG-tRNA$^{AsnE2}_{GGU}$,<br>50 µM ^{ClAc}Y-tRNA$^{fMet}_{CAU}$	37°C	60 min
Pep7-A^{S}-Br	mR7	D, G, K, M, V, Y	50 µM BrvG-tRNA$^{AsnE2}_{GGU}$,<br>50 µM Ala^{S}-tRNA$^{GluE2}_{GUG}$	37°C	30 min

References

1 Wang, C., Tobrman, T., Xu, Z. & Negishi, E. Highly regio- and stereoselective synthesis of (Z)-trisubstituted alkenes via propyne bromoboration and tandem Pd-catalyzed cross-coupling. *Org. Lett.* **11**, 4092-4095 (2009).

2 Hall, D., Gernigon, N., Al-Zoubi, R. & Thornton, P. D. Boronic acid catalysts and methods of use thereof for activation and transformation of carboxylic acids. WO 2012109749 (2012).

3 Petasis, N. A. & Zavialov, I. A. A new and practical synthesis of α-amino acids from alkenyl boronic acids. *J. Am. Chem. Soc.* **119**, 445-446 (1997).

4 Goto, Y., Katoh, T. & Suga, H. Flexizymes for genetic code reprogramming. *Nat. Protoc.* **6**, 779 (2011).
