# Supplementary material for: Posttranslational chemical installation of azoles into translated peptides
Source: Nat Commun. 2021 Jan 29;12:696. doi: 10.1038/s41467-021-20992-0 (PMC7846737; doi:10.1038/s41467-021-20992-0)
Supplement: Supplementary file 2 — Reporting Summary [file 41467_2021_20992_MOESM2_ESM.pdf]

nature research

Corresponding author(s): Hiroaki Suga, Yuki Goto

Last updated by author(s): Oct 28, 2020

Reporting Summary

Nature Research wishes to improve the reproducibility of the work that we publish. This form provides structure for consistency and transparency in reporting. For further information on Nature Research policies, see our Editorial Policies and the Editorial Policy Checklist.

Statistics

For all statistical analyses, confirm that the following items are present in the figure legend, table legend, main text, or Methods section.

n/a	Confirmed	
☒	☐	The exact sample size (n) for each experimental group/condition, given as a discrete number and unit of measurement
☒	☐	A statement on whether measurements were taken from distinct samples or whether the same sample was measured repeatedly
☒	☐	The statistical test(s) used AND whether they are one- or two-sided<br>*Only common tests should be described solely by name; describe more complex techniques in the Methods section.*
☒	☐	A description of all covariates tested
☒	☐	A description of any assumptions or corrections, such as tests of normality and adjustment for multiple comparisons
☐	☒	A full description of the statistical parameters including central tendency (e.g. means) or other basic estimates (e.g. regression coefficient) AND variation (e.g. standard deviation) or associated estimates of uncertainty (e.g. confidence intervals)
☒	☐	For null hypothesis testing, the test statistic (e.g. F, t, r) with confidence intervals, effect sizes, degrees of freedom and P value noted<br>*Give P values as exact values whenever suitable.*
☒	☐	For Bayesian analysis, information on the choice of priors and Markov chain Monte Carlo settings
☒	☐	For hierarchical and complex designs, identification of the appropriate level for tests and full reporting of outcomes
☒	☐	Estimates of effect sizes (e.g. Cohen's d, Pearson's r), indicating how they were calculated

Our web collection on statistics for biologists contains articles on many of the points above.

Software and code

Policy information about availability of computer code

Data collection	Gel electrophoresis data was acquired on a GE Healthcare FLA 7000 under control of FLA 7000 software v.1.2. MALDI-MS data was acquired on a Bruker Daltonics ultrafleXtreme instrument under control of flexControl v.3.4. LC-ESI-MS data was acquired on a Waters Xevo G2-XS QTof instrument under control of MassLynx v.4.1.
Data analysis	Gel electrophoresis data was analyzed in ImageQuant TL v.8.1. MALDI-MS data was analyzed in flexAnalysis v.3.4. LC-ESI-MS data was analyzed in MassLynx v.4.1.

For manuscripts utilizing custom algorithms or software that are central to the research but not yet described in published literature, software must be made available to editors and reviewers. We strongly encourage code deposition in a community repository (e.g. GitHub). See the Nature Research guidelines for submitting code & software for further information.

Data

Policy information about availability of data

All manuscripts must include a data availability statement. This statement should provide the following information, where applicable:

- Accession codes, unique identifiers, or web links for publicly available datasets
- A list of figures that have associated raw data
- A description of any restrictions on data availability

Primer sequences, DNA template assembly schemes, characterization of BrvG-DBE, gel electrophoresis and MALDI-TOF-MS data to demonstrate genetic code reprogramming using BrvG, MS/MS identification of Pep1-Br, and summary of all LC-MS data discussed in this paper are available in Supplementary Information. Other results are available from the corresponding authors upon reasonable request.

Field-specific reporting

Please select the one below that is the best fit for your research. If you are not sure, read the appropriate sections before making your selection.

[x] Life sciences [ ] Behavioural & social sciences [ ] Ecological, evolutionary & environmental sciences

For a reference copy of the document with all sections, see nature.com/documents/nr-reporting-summary-flat.pdf

Life sciences study design

All studies must disclose on these points even when the disclosure is negative.

Sample size	For quantification of FIT-expressed Pep1-Br, n=3 independent samples were chosen to test the reproducibility of the experiment.
Data exclusions	No data was excluded.
Replication	The chemical conversion of Pep1-Br to Pep1-Oxz was successfully repeated by three distinct persons at least 15 times in total. Quantification of the in vitro expressed peptides was repeated three times and their averaged values were reported. Syntheses of most of azole-containing peptides shown in the article were successfully repeated at least two times each, except for Pep2(Glu), Pep2(Tyr), and Pep4-Oxz-G-Oxz, which were synthesized once.
Randomization	It is not relevant to this study since no experiment was carried out on humans or animals.
Blinding	Experimental designs did not require and were incompatible with blinding. No experiments requiring blinding were performed.

Reporting for specific materials, systems and methods

We require information from authors about some types of materials, experimental systems and methods used in many studies. Here, indicate whether each material, system or method listed is relevant to your study. If you are not sure if a list item applies to your research, read the appropriate section before selecting a response.

Materials & experimental systems

n/a	Involved in the study	
[x]	[ ]	Antibodies
[x]	[ ]	Eukaryotic cell lines
[x]	[ ]	Palaeontology and archaeology
[x]	[ ]	Animals and other organisms
[x]	[ ]	Human research participants
[x]	[ ]	Clinical data
[x]	[ ]	Dual use research of concern

Methods

n/a	Involved in the study	
[x]	[ ]	ChIP-seq
[x]	[ ]	Flow cytometry
[x]	[ ]	MRI-based neuroimaging
